# On the mechanisms underlying activation and reversal of high altitude‐induced pulmonary hypertension in humans – Another piece in the pulmonary puzzle

**DOI:** 10.1113/EP090967

**Published:** 2022-11-21

**Authors:** Marc Moritz Berger, Damian Miles Bailey

**Affiliations:** ^1^ Department of Anesthesiology and Intensive Care Medicine University Hospital Essen University Duisburg‐Essen Essen Germany; ^2^ Neurovascular Research Laboratory Faculty of Life Sciences and Education University of South Wales Pontypridd UK

**Keywords:** endothelin, high altitude, hypoxia, hypoxic pulmonary vasoconstriction, nitric oxide, reactive oxygen species, Sherpa, vascular remodelling

1

More than 140 million people live above 2,500 m a.s.l. (Penaloza & Arias‐Stella, 2007), where the partial pressure of inspired O_2_ is reduced because of a reduction in barometric pressure, leading to constriction of precapillary resistance vessels in the lung. This mechanism, known as hypoxic pulmonary vasoconstriction (HPV), is a highly conserved adaptive response to optimize ventilation–perfusion matching and alveolar gas exchange by diverting blood flow from poorly ventilated to better‐oxygenated areas of the lung. Global alveolar hypoxia leads to sustained vasoconstriction and consequent elevation in pulmonary vascular resistance (PVR) and pulmonary artery pressure (PAP). However, exaggerated HPV in the acute setting can predispose to high‐altitude pulmonary oedema, a life‐threatening non‐cardiogenic form of pulmonary oedema that can develop in non‐acclimatized healthy individuals who ascent too high, too fast (Bartsch & Swenson, 2013).

Pulmonary vasoconstriction in acute hypoxia occurs in two phases (Figure 1). In the first phase, hypoxia increases formation of free radicals and associated reactive oxygen species (ROS) that lead to an increase in intracellular calcium and contraction of pulmonary vascular smooth muscle cells (Smith & Schumacker, 2019). In the second phase, vasoconstriction is maintained by the ʻdouble whammy’ of a sustained reduction in vascular NO bioavailability combined with an increase in endothelium‐derived vasoconstrictors (Bailey et al., 2010; Dunham‐Snary et al., 2017). When the hypoxic stimulus is brief (a few hours), HPV is completely reversible upon restoration of normoxia or administration of supplemental O_2_. Intriguingly, however, as the duration of hypoxia increases, the reversibility of HPV in response to supplemental O_2_ progressively decreases. This can be explained by vascular remodelling leading to structural changes in pulmonary vessels that increase vascular stiffness, decrease the luminal diameter of arteries and increase resistance to blood flow. These processes are considered to be mediated predominantly by hypoxia‐inducible factor‐1α (HIF‐1 α), which is modulated by endothelin‐1 and NO, and stabilized by hydrogen peroxide arising from superoxide anion dismutation (Pisarcik et al., 2013; Pugh, 2016; Smith & Schumacker, 2019). In addition, ROS can amplify the proliferation of fibroblasts in the adventitial layer and promote their expansion between the endothelium and the neointima (Aggarwal et al., 2013). Pulmonary hypertension places an increased pressure load on the right ventricle that, if left untreated, can progress to right heart failure and, ultimately, premature death.

**FIGURE 1:**
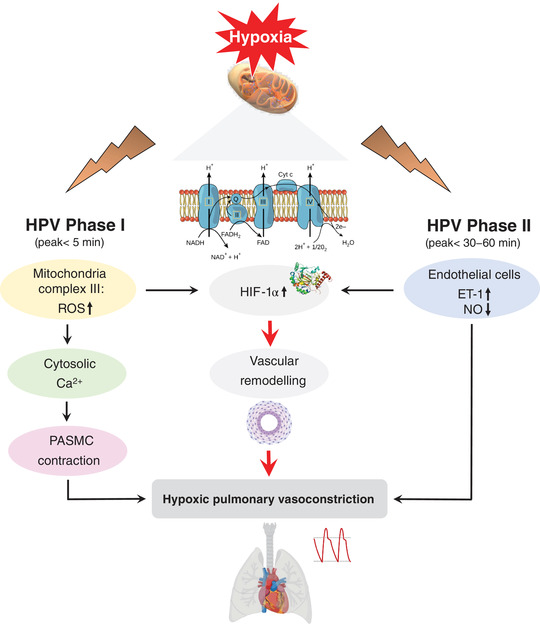
Phases and mechanistic pathways underlying HPV in humans. In the first phase, hypoxia increases the production of free radicals and associated ROS (primarily superoxide anions and hydrogen peroxide) at complex III of the mitochondrial electron transport chain. After being released to the cytosol, superoxide is converted to hydrogen peroxide, which activates downstream targets that induce an increase in intracellular calcium concentration and contraction of pulmonary vascular smooth muscle cells (Smith & Schumacker, 2019). Vasoconstriction is subsequently maintained by a reduction in vascular NO bioavailability combined with an elevation in the circulating concentration of endothelium‐derived vasoconstrictors (Bailey et al., 2010; Dunham‐Snary et al., 2017). If HPV is maintained, HIF‐1α activates a complex signalling cascade, ultimately inducing pulmonary vascular remodelling and fixation of HPV (Smith & Schumacker, 2019). Abbreviations: ET‐1, endothelin‐1; HIF‐1α, hypoxia‐inducible factor‐1α; HPV, hypoxic pulmonary vasoconstriction; PASMCs, pulmonary arterial smooth muscle cells; ROS, reactive oxygen species

The time course at high altitude over which HPV transitions from being acutely reversible by O_2_ supplementation (hyperoxia) to irreversible is poorly characterized. It is also unknown how quickly the pulmonary vasculature ‘regains’ its responsiveness after returning to the more O_2_‐rich climes experienced at low altitude. In this issue of *Experimental Physiology*, Subedi et al. (2022) investigated how HPV and the response to supplemental O_2_ change over time during ascent to high altitude. They compared non‐acclimatized lowlanders against two separate groups of Sherpas who either stayed at their home of residence (>3,500 m a.s.l.) or left to spend 5–15 days de‐acclimatizing at lower altitudes in Kathmandu (∼1,300 m). The authors demonstrated that both non‐acclimatized lowlanders and Sherpas exhibited a reduction in PVR and PAP in response to supplemental O_2_ during ascent to 5,050 m over a duration of 8–10 days. However, after an additional 14 days at 5,050 m, the pulmonary vascular responsiveness to O_2_ was almost abolished and comparable to that exhibited by fully acclimatized Sherpas. Of note, after ∼26 days at 5,050 m, more severe hypoxia (or hyperoxia) had no additional effect on HPV in lowlanders or either group of Sherpas, implying structural changes in the pulmonary vasculature over the longer term.

The study by Subedi et al. (2022) concurs with Luks et al. (2017), who demonstrated that the acute responsiveness of the pulmonary circulation to either hypoxia or hyperoxia was not impaired during 12–13 days of progressive ascent from sea level to 5,300 m, suggesting that the pulmonary circulation maintained its overall responsiveness to changes in inspired O_2_. However, according to the findings by Subedi et al. (2022), 14 days at 5,050 m were required almost as a ‘threshold duration’ to ablate pulmonary vasculature reactivity to supplemental O_2_ fully. This finding is supported by Maggiorini et al. (2001), who demonstrated that in mountaineers with and without prior susceptibility to high‐altitude pulmonary oedema, hyperoxia failed fully to reverse the increase in PAP observed after 48 h at 4,559 m. Collectively, these findings indicate that vascular remodelling occurs early after exposure to altitudes >4,500 m a.s.l. and that it is (almost) complete after ∼2 weeks of continued residence at ∼5,000 m.

Unlike most forms of pulmonary hypertension, which are known to be progressive and life‐limiting, the vascular changes induced by chronic hypoxia are reversible. However, the minimum length of stay at low altitude that is required to allow the pulmonary vasculature to become responsive to O_2_ again is not well defined. Despite no amelioration in response to acute hyperoxia, Sime et al. (1971) demonstrated normalization of PVR and PAP in Peruvian Andeans after >2 years of residence at sea level. In addition, Anand et al. (1990) observed full reversal of cardiomegaly and normalization of PVR and PAP in Indian soldiers 12–16 weeks after descending from >5,800 to 300 m. An interesting finding by Subedi et al. (2022) is how fast the pulmonary vascular responsiveness to hyperoxia was (partly) restored in the group of Sherpas who descended from >3,800 m to low altitude. After only 5–15 days (median 7 days!) at 1,300 m, pulmonary vascular reactivity during re‐ascent to altitude was comparable to that observed in the unacclimatized lowlanders. These findings are in agreement with Hilty et al. (2016), who reported that PAP after 4 weeks residence at 3,454 m in lowlanders was fully reversible within 1 week after return to sea level. However, by their own admission, Subedi et al. (2022) failed to correct for alterations in haematocrit or to measure changes in systemic/transpulmonary redox homoeostasis, which are collectively known to impact haemostasis and associated vascular reactivity (Bailey et al., 2010; Fall et al., 2018). Food for thought in future studies!

From a clinical translational perspective, exposure to terrestrial high altitude can provide unique insight into the mechanisms underlying the long‐term impact of (chronic) hypoxia on the pulmonary vasculature, including the molecular nuances that underlie structural remodelling. The approach of investigating native lowlanders exposed to high altitude and native highlanders offers the advantage that a relatively homogeneous and well‐characterized population can be studied. It also allows better dissection of the effect of environmental and genetic factors on (mal)adaption to high altitude. However, although similarities might exist between the pathological features of high altitude‐induced pulmonary hypertension and other forms of hypoxia‐induced pulmonary hypertension (e.g., owing to chronic obstructive pulmonary disease), the extent of overlap in the pathological mechanisms remains unclear. Understanding the integrated mechanisms and temporal kinetics underlying the pulmonary vascular response to terrestrial high‐altitude hypoxia has the potential to provide unique insight into (terrestrial) treatment of pulmonary hypertension. Subedi et al. (2022) can be congratulated for their study, which adds another piece to the pulmonary puzzle that helps us to gain a better understanding of the development and reversibility of high altitude‐induced pulmonary hypertension.

## AUTHOR CONTRIBUTIONS

Marc M. Berger and Damian M. Bailey conceived the idea and wrote the first draft of the manuscript. Marc M. Berger and Damian M. Bailey approved the final version submitted for publication and agree to be accountable for all aspects of the work in ensuring that questions related to the accuracy or integrity of any part of the work are appropriately investigated and resolved. Both persons designated as authors qualify for authorship, and all those who qualify for authorship are listed.

## CONFLICT OF INTEREST

D.M.B. is Chair of the Life Sciences Working Group and member of the Human Spaceflight and Exploration Science Advisory Committee to the European Space Agency and is a member of the Space Exploration Advisory Committee to the UK Space Agency. D.M.B. is affiliated to the companies FloTBI, BrainEx and OrgEx focused on the technological development of novel biomarkers of brain injury in humans.
